# Familiarity affects social network structure and discovery of prey patch locations in foraging stickleback shoals

**DOI:** 10.1098/rspb.2014.0579

**Published:** 2014-08-22

**Authors:** N. Atton, B. J. Galef, W. Hoppitt, M. M. Webster, K. N. Laland

**Affiliations:** 1 School of Biology, University of St Andrews, Harold Mitchell Building, Fife KY16 9TH, UK; 2 Department of Psychology, Neuroscience and Behaviour, McMaster University, Hamilton, Ontario, Canada L8S 4K1; 3 Animal and Environment Research Group, Anglia Ruskin University, Cambridge CB1 1PT, UK

**Keywords:** innovation, social information, social learning, social network, social organization

## Abstract

Numerous factors affect the fine-scale social structure of animal groups, but it is unclear how important such factors are in determining how individuals encounter resources. Familiarity affects shoal choice and structure in many social fishes. Here, we show that familiarity between shoal members of sticklebacks (*Gasterosteus aculeatus*) affects both fine-scale social organization and the discovery of resources. Social network analysis revealed that sticklebacks remained closer to familiar than to unfamiliar individuals within the same shoal. Network-based diffusion analysis revealed that there was a strong untransmitted social effect on patch discovery, with individuals tending to discover a task sooner if a familiar individual from their group had previously done so than if an unfamiliar fish had done so. However, in contrast to the effect of familiarity, the frequency with which individuals had previously associated with one another had no effect upon the likelihood of prey patch discovery. This may have been due to the influence of fish on one another's movements; the effect of familiarity on discovery of an empty ‘control’ patch was as strong as for discovery of an actual prey patch. Our results demonstrate that factors affecting fine-scale social interactions can also influence how individuals encounter and exploit resources.

## 1. Introduction

The fine-scale social organization of animal groups is shaped by a range of individual-level variables, including internal state, pathology, parasite load, active preferences for certain group mates or near-neighbours and other phenotypic characters [1–4]. The frequency and nature of interactions between individual group members is likely to affect the pattern and rate with which information and innovations are transmitted through populations [5]. For researchers interested in understanding the diffusion of information, accounting for factors that shape local, fine-scale group structure is therefore essential.

In many species of fishes, a preference for associating with familiar individuals has been shown to strongly affect social organization [6,7]. Broadly speaking, familiarity operates via at least two mechanisms [8]. The first is based upon learned recognition of other individuals. After a period of continuous interaction, fish come to remember the identity of their shoalmates and prefer to group with familiar individuals over other conspecifics with whom they have not previously interacted (e.g. [9]; reviewed by Griffiths & Ward [7]). Familiarity also occurs via a more general mechanism based upon self-referent matching, whereby fish prefer to associate with individuals that have recently eaten the same prey, or occupied similar habitat types, as themselves [8,10–15]. While the precise mechanism underlying self-referent recognition is not fully understood, it potentially operates via diet- or habitat-derived amino acids or other metabolites released via the gills (in freshwater), or through the urine or epidermal mucus of the fish [10,16–19].

Learned familiarity has been shown to play a role in directing the transmission of information between guppies (*Poecilia reticulata*) under binary choice conditions. In a study by Lachlan *et al*. [20], individual adult ‘focal’ guppies were presented with two diverging shoals of conspecifics, one consisting of familiar and the other of unfamiliar individuals. When given the choice of following familiar or unfamiliar shoals when each swam to an opposite end of a tank, focal guppies were more likely to follow the shoal comprising familiar rather than unfamiliar fish. Following can lead to information acquisition and social learning if, for example, following leads naive individuals to a food source [21,22]. A further study using guppies as subjects [23] tested the hypothesis that the fish would learn more effectively from familiar than unfamiliar demonstrators trained to swim a particular route to a food source. Trained demonstrators were placed with small shoals of untrained observers. Untrained individuals were more likely to discover and subsequently learn the route to the food source when demonstrators were familiar to them than when they were unfamiliar, suggesting that familiarity between individuals can influence social learning, with individuals learning more effectively from familiar conspecifics. In nature, given the fission–fusion dynamics occurring in many social systems [24], it seems likely that groups will contain individuals both familiar and unfamiliar to each group member.

Until relatively recently, statistical tools able to quantify the effects of factors promoting differences in fine-scale social organization (such as differences in familiarity) upon patterns of information transmission in free-ranging groups did not exist. The development of network-based diffusion analysis (NBDA) [25,26], allowing non-random transmission of information to be detected, permits such factors to be accounted for quantitatively. Indeed, recent research has demonstrated that NBDA is a useful method for quantifying the diffusion of learned behavioural innovations through populations of fishes, birds and mammals in both the laboratory and the wild [27–30].

In this study, we sought to determine the influence of fine-scale social organization on patterns of information diffusion, using NBDA of the foraging behaviour of groups of threespine sticklebacks (*Gasterosteus aculeatus*) that contained both familiar and unfamiliar individuals. Sticklebacks are an appropriate study system for examining the role of familiarity in modulating the diffusion of information through social groups. Sticklebacks are competent social learners; Coolen *et al*. [31], van Bergen *et al*. [32], Laland *et al*. [33] and Frommen *et al*. [34] have previously shown them to be useful for investigating the role of social transmission and social learning in foraging behaviour, and they have served as a model organism for network-based diffusion analyses [29,30]. Sticklebacks detect familiarity through both learned recognition [35] and recognition of habitat- and diet-derived cues [11–13,15].

Here, our primary aim was to determine the role, if any, of familiarity in driving directed transmission of information. The precise mechanism by which familiarity occurred was of less concern. Consequently, to maximize the differentiation between familiar and unfamiliar fish, we used a protocol that allowed for familiarity to occur via both learned and resource-derived recognition. We achieved this by housing groups of fish in separate groups and feeding them on different diets for several weeks prior to testing them. This provided the opportunity for both learned familiarity [9] and recognition based upon diet-derived chemical cues [8] to occur. We adopted the approach of Atton *et al*. [29]; groups of sticklebacks were placed into an arena and allowed to search for and feed upon prey placed within novel feeders. We tested the following predictions: (i) that the social network structure of groups of sticklebacks would be influenced by familiarity, with more frequent patterns of association between familiar than between unfamiliar fish; (ii) that information would diffuse via association networks, with greater likelihood of transmission between more strongly compared with weakly associated individuals; and (iii) that individuals would be more likely to acquire information about the location of feeders and the way to access them from familiar than from unfamiliar individuals.

## 2. Material and methods

### (a) Subjects and treatment groups

In April 2012, we used wire-mesh cage traps to capture 80 three-spined sticklebacks in the Kinnessburn, a small stream in St Andrews, UK (56.3349° N, 2.7885° W). We transported the fish to our laboratory where they were held in two groups of 40 fish in 90 l tanks at a temperature of 8°C. We fed the fish daily with frozen bloodworms (*Chironomus* sp. larvae) for two weeks immediately following capture.

For the month following this initial holding period, we fed one group exclusively on *Artemia* and the other exclusively on *Tubifex*. Hereafter, fish taken from the same holding tank are referred to as ‘familiar’ and fish from different holding tanks as ‘unfamiliar’.

We formed seven replicate groups from these 80 fish, each containing 10 individually marked fish [36], measuring 35–45 mm in length. We did not use individuals displaying signs of nuptial coloration or gravidity, as reproductive state has been shown to affect an individual's reliance on social information in sticklebacks [35]. Each group of 10 fish contained five fish from the *Artemia*-fed treatment and five from the *Tubifex*-fed treatment, selected from their holding tanks so that individuals in each group were size matched to within 4 mm.

### (b) Test arena and procedure

**Figure 1.**
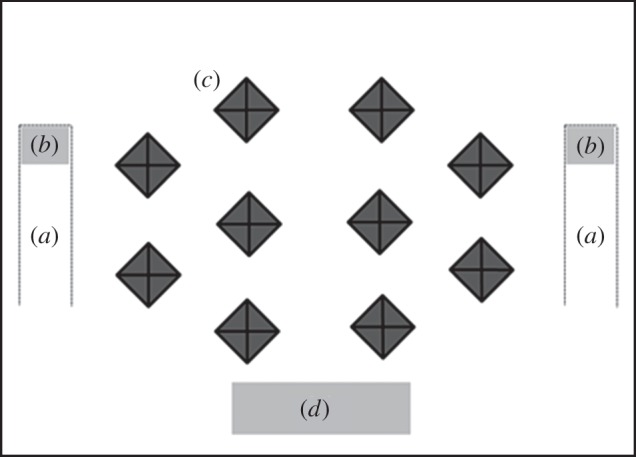
The experimental arena, containing two feeding tubes (*a*). Each of these held a prey patch (*b*), while the other was empty and served as a control patch. Ten plastic pyramids (*c*) were used to provide structural complexity. An empty control patch (*d*) was used to estimate for untransmitted social effects on patch entry. See main text for full details.

We tested each group separately in a rectangular black test tank measuring 60 × 80 cm. To ensure that vertical distance within the water column between individuals did not greatly confound estimates of inter-individual distances (described below), we filled the test tank with filtered tap water to a depth of only 5 cm. The test tank had a gravel substrate 1 cm in depth, and 10 black, pyramid-shaped obstacles (measuring 10 cm square at the base and 6 cm high) placed at regular intervals throughout the tank (figure 1) to provide a degree of structural complexity while allowing the experimenter to view all fish at all times, from above using a video camera. The test tank was located within a shelter to minimize outside disturbance. We recorded each trial through a small hole in the roof of the shelter using a Canon HG20 video camera fixed 1.2 m above the test tank. Two 60 W fluorescent strip lights provided illumination.

In the second phase of the trial described below, we provided bloodworm prey in two identical feeders. Each feeder consisted of a transparent cylindrical tube measuring 24 cm in length and 7 cm in diameter, open at only one end, placed horizontally on the gravel substrate at each end of the test tank [29]. We inserted 15 ml of defrosted bloodworms suspended in water into the closed end of each tube, which had 15 small holes (2 mm diameter) allowing chemical cues from bloodworms to escape. The entrance to the open, accessible end of the tube was clearly marked with black electrical tape placed around its circumference. Consequently, although fish could see and smell food at both ends of each tube, they could gain access to food only by entering a tube through its open end. Fish were tagged 2 h before the trial began. The tags consisted of 5 mm diameter PVC discs weighing approximately 10 mg [29,30]. These were pierced at the centre using a 0.4 mm needle and placed over the first dorsal spine of the fish. Each tag carried a unique colour and symbol combination that could be read from the video recording of the trial. This procedure is non-invasive and has been shown to have no effect on the shoaling behaviour of the fish [36]. At the end of the trial, the tags were removed. A group of 10 fish was placed in the test tank and allowed to settle for 15 min. A trial then commenced. The trial had two phases: an association phase, used to quantify social network structure, and a foraging phase, from which we extracted data to perform the NBDA. The association phase lasted 120 min, during which we point sampled the shoaling behaviour of the fish at 6 min intervals, giving a total of 20 observations for each group. A pair of individuals were classed as shoaling if they were within four body lengths of one another (defined as the mean body length of the groups’ members) from head to head (a distance generally accepted as indicative of shoaling in fishes [37]). We used a ‘gambit of the group approach’ [38,39], so that a string of fish connected by less than two body lengths were all assumed to be associating with one another. We used these data to create an association matrix for each group of 10 fish based upon the proportion of point samples that each fish was observed to be within four body lengths of each of the other fish in its group.

Following the 120 min association phase, we carefully introduced the foraging tasks and filmed the group for a further 45 min, after which the trial ended and we removed all fish from the experiment. We recorded both the latency with which each individual first discovered each task (defined as occurring when an individual was seen striking at food through the transparent tube) and the latency with which each individual first solved each task (defined as consuming food within the tube). We scored only the first 20 min of video footage after the first fish in each group solved each task. None of the food patches were completely exhausted during this 20 min period.

In addition to recording the latency to discovery and solution of the tasks, we also recorded the latency with which each fish entered an arbitrary area (measuring 20 × 10 cm) within the tank. This control location contained no food and no distinctive topographical features. In our analysis, we compared the strength of social effects on the order in which fish ‘discovered’ the empty control patch location with the strength of social effects on the order in which fish discovered the foraging tasks. This comparison allowed us to distinguish social transmission from other processes that might result in a superficially similar pattern of acquisition [30].

### (c) Testing for effects of familiarity upon association preferences

We used a randomization test to determine whether fish preferred to associate with familiar or unfamiliar fish. For each group, we constructed a binary matrix indicating whether each pair of fish were familiar (1) or not (0). We then ran a simple regression with the values from the upper triangle of each association matrix as the response, and the upper triangle of the habitat matrix as the predictor. The coefficient of the slope was taken as the test statistic. We generated a null distribution by randomizing the rows and columns within each habitat matrix and recalculating the test statistic 100 000 times. The *p*-value was taken to be (1 + number of the null distribution > test statistic)/100 001 [40,41]. Analyses were conducted in the R statistical environment v. 2.15.3 [42].

### (d) Network-based diffusion analysis

To analyse the data from the foraging phase of the experiment, we used NBDA [25]—specifically, the order of acquisition diffusion analysis (OADA) variant of NBDA [26]. OADA can be used to determine whether the order in which subjects discover and/or solve a task is correlated with different social networks, each representing a different hypothesis as to the pathway of transmission. We used the multi-state version of OADA (developed by Atton *et al*. [29]), which models multiple options that can be used to solve a task (in this case, the two versions of the feeder task), allowing us to both distinguish option-specific and cross-option social effects, and tease apart social effects on the rate at which fish (i) discover each option and (ii) solve each option once they have discovered it (see the electronic supplementary material for a full model specification).

We compared the predictive power of three social networks: (i) one reflecting patterns of association observed during the association phase of the experiment, thus testing the hypothesis that the rate of social transmission from individual A to individual B is proportional to the association between them; (ii) one in which there were binary connections only between familiar individuals (the ‘familiar network’), thus testing the hypothesis that information is transmitted only between familiar individuals; and (iii) one in which there were binary connections between all individuals in the same group (the ‘homogeneous network’), thus testing the hypothesis that information is transmitted homogeneously between all members of the group regardless of their familiarity [42]. We also fitted a model with different social effects between fish that were familiar and unfamiliar to test the hypothesis that there was a social effect between all individuals, but that it was stronger between fish that were familiar than between fish that were unfamiliar to one another [43]. For each social network, we also considered both additive and multiplicative models for the interaction between social effects and asocial learning (see [26] for details).

We included ‘holding tank’ and ‘group’ as factors in our analyses to examine the possibility that individuals from each of the holding tanks and/or experimental groups might differ systematically in their rates of asocial discovery or solving of the foraging tasks. We also included factors allowing for an overall bias for left or right feeder and for an effect of having discovered/solved one option on the rate of discovery/solving the other option. Models were fitted with each combination of network, additive versus multiplicative model and presence/absence of other factors, excluding group (we considered social effects and group differences in asocial rates of learning as alternative explanations for differences between groups, and consequently only included ‘group’ in models with no social network).

We used a model averaging approach based on Akaike's information criterion corrected for sample size (AICc) to estimate the effects of each predictor variable, accounting for model selection uncertainty, and to quantify the relative support for each network/variable using summed Akaike weights [44] (see electronic supplementary material). Unconditional confidence intervals were calculated using the profile likelihood technique corrected for model selection uncertainty suggested by Burnham & Anderson [44].

## 3. Results

### (a) Effects of familiarity upon association preferences

**Figure 2.**
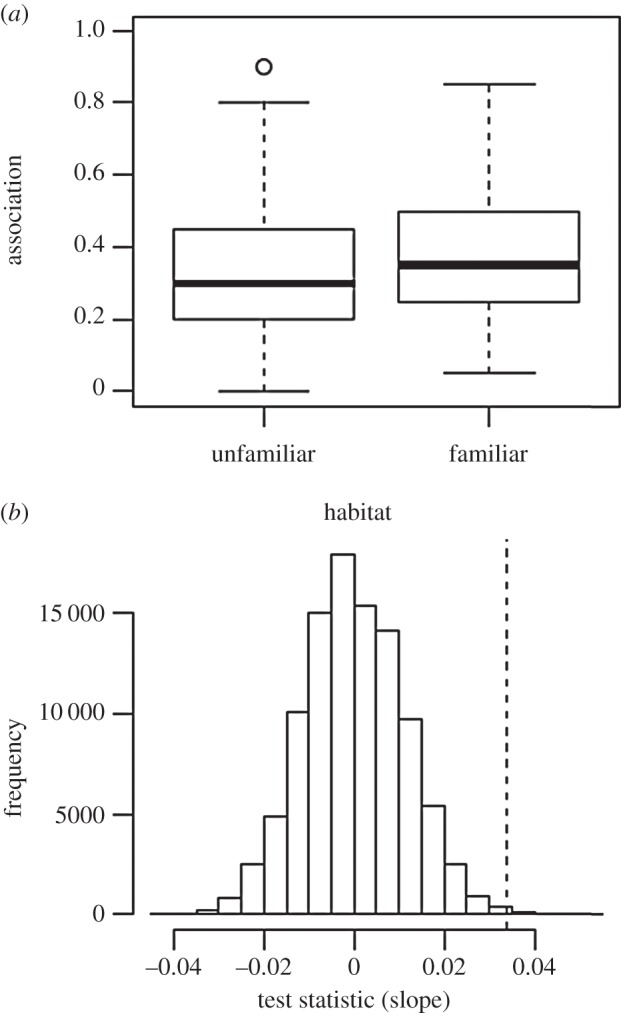
(*a*) Box plot of association strengths for familiar and unfamiliar fish backgrounds. (*b*) Histogram showing the null distribution from the randomization test. The dashed line shows the observed test statistic.

There was a significant difference in association between familiar and unfamiliar fish backgrounds (*p* = 0.001; means: familiar = 0.366; unfamiliar = 0.332), though there was considerable variability within each category (figure 2).

### (b) Network-based diffusion analysis

#### (i) First discovery

**Figure 3.**
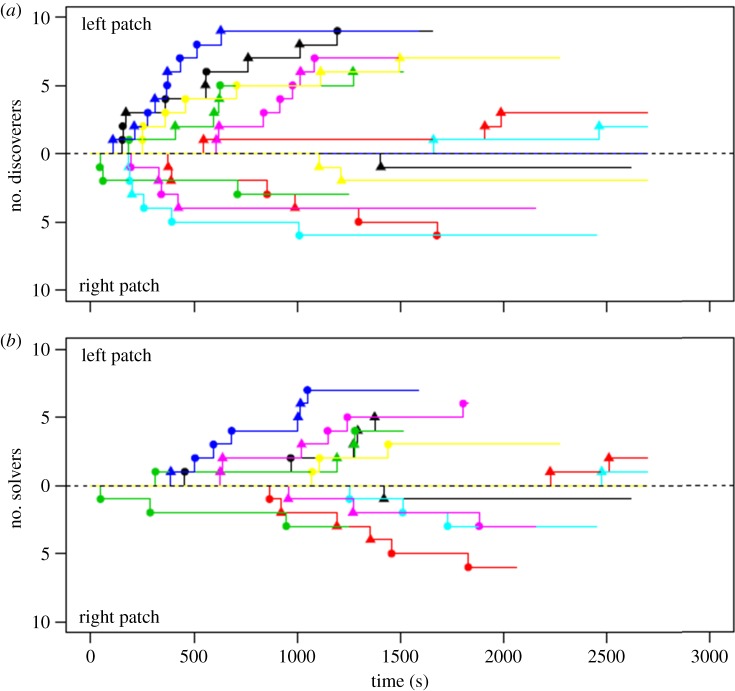
The diffusion curves for the times of (*a*) first discovery and (*b*) first solving, showing times for both the left- and right-hand options. Each colour represents a different group. Circles represent individuals held together and fed the *Tubifex* diet, and triangles represent individuals held together and fed the *Artemia* diet. Individuals represented by the same symbols are considered to be familiar to one another. (Online version in colour.)

There were no trials in which all individuals within a group discovered both options, with the number of discoverers of each option ranging from 0 to 9. A minimum of seven individuals discovered one or the other of the options, with a total of 60 first discoveries across both individuals and options (figure 3). Diffusion curves for discovery time (figure 3) generally reveal a rapid increase in number of individuals discovering one option after initial discovery of that same option by another within a group.

#### (ii) Support for effect of different social networks on discovery rate

In all seven groups, at least four fish solved one task within the allotted 20 min following first solution within that group. No scrounging was observed, as solvers did not move food items outside the tube. There were a total of 39 first solves across all individuals and options.

There was strongest support for the familiarity network influencing the rate at which individuals discovered the tasks (Akaike weight of 86.2% for multiplicative model), with fish being more likely to discover a task if familiar fish had already done so, but not if unfamiliar individuals had discovered the task (see figure 3 and table 1). A homogeneous network received little support, with an Akaike weight of 5.4%. The association network also received very little support (Akaike weight = 1.8%), as did a model with no social effect (Akaike weight = 0.0%). A multiplicative model with the familiarity network was therefore used to estimate the effects reported in §3*b*(iii) (using model averaging across other predictor variables).

**Table 1.** A comparison of the support (based on Akaike weight) for familiarity and homogeneous effects on the discovery of the foraging tasks.

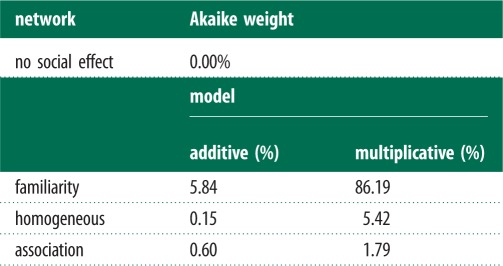

We also fitted a multiplicative model with different social effects between familiar (*s*_same_) and unfamiliar fish (*s*_diff_), allowing us to assess the difference between the two. *s*_same_ was estimated at 1.80 (95% CI = (0.68, 4.52)) and *s*_diff_ was estimated at 0.20 (95% CI = (0, 1.18)), giving an estimated difference of 1.60 (95% CI = (0.46, 3.96)), providing evidence of a stronger social effect on discovery between familiar than between unfamiliar individuals. In sum, for each individual, the increase in discovery rate for each informed familiar individual in its group relative to the rate of asocial discovery was estimated to be 1.6 units greater than the effect of an informed unfamiliar individual.

#### (iii) Estimates of effects on discovery rate

Both estimates from the NBDA and support for each variable are shown in table 2. An explanation of the method used to calculate support for each variable (Akaike's information criterion) can be found in [29]. There was strong evidence (total Akaike weight = 100%) that being well connected to familiar individuals who had already discovered an option increased the probability that a naive individual would be the next to discover that option, providing clear evidence of a social effect on task discovery. The magnitude of this effect was estimated to be a linear increase of 1.5 times (95% CI = (0.64, 3.5)) the average asocial rate of discovery for every informed familiar individual. However, evidence for social transmission of the patch location was unsubstantial, with the contrast between real and control food patches (*S*_R_ − *S*_C_) estimated at 0.716 (95% CI: (−1.14, 2.75)).

**Table 2.** Two option NBDA results, showing support for factors affecting task discovery by naive individuals.

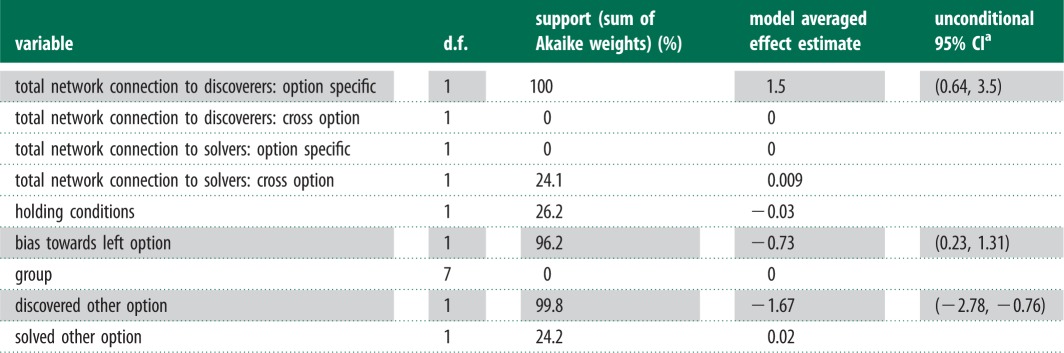

^a^Shaded cells indicate that there was more support for an effect than against (more than 50%). Social effects are estimated relative to the mean rate of asocial discovery (e.g. a value of 1.5 signifies that an average individual with one unit of total association to discoverers of an option is 1.5 times faster to discover the same option than an average individual with no connections to discoverers of that option). Unconditional 95% confidence intervals were calculated using a computationally intensive profile likelihood procedure (see the electronic supplementary material of [29]), so we only calculated these for variables with support of more than 50%.

The finding that effects of familiarity at real patches were not greater than those at the control patch suggests that the social effect on discovery may be a result of associated individuals encountering the task at approximately the same time because of their influence on each other's movements, rather than individuals transmitting the location of a real patch once they had discovered it.

There was no evidence that being well connected to solvers of an option facilitated discovery of that option (over and above their effect as individuals that had discovered that option), and no evidence against an effect of connectedness to discoverers generalizing across options (total Akaike weight = 0.0%). There was also little support for the hypothesis that individuals were more likely to discover an option next if they were well connected to individuals who had solved the other task option than if they were not so connected (total Akaike weight = 24.1%). There was little evidence of a difference in the rate of discovery between individuals from each of the two holding tanks. There was, however, strong evidence that individuals that had discovered one option were less likely to discover the other (Akaike weight = 99.8%). There was also strong evidence of a bias towards the left-hand task (Akaike weight = 96.2%).

#### (iv) Support for effect of different social networks on solving rate

There is most support for an effect of the familiarity network (Akaike weight: additive = 35.3%; multiplicative = 28.5%) on the rate at which individuals solve the tasks (table 3), with fish being more likely to solve a task if familiar fish had already done so, but not if unfamiliar individuals had solved the task (figure 3); however, this evidence is not particularly strong: the model with no social effect had an Akaike weight of 8.4%. There was little support for the homogeneous network (Akaike weight: additive model = 10.0%; multiplicative model = 8.9%) or association network (Akaike weight: additive model = 4.42%; multiplicative model = 4.38%), suggesting a familiarity-based social effect is the most likely. The additive familiarity network was used to estimate the effects below.

**Table 3.** A comparison of the support (based on Akaike weight) for familiarity and homogeneous effects on the solving of the foraging tasks.

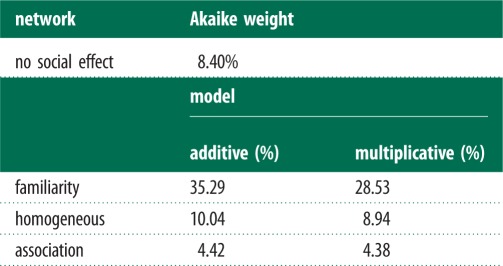

#### (v) Estimates of effects on solving rate

As confirmed by the NBDA, diffusion curves for the solving times of the foraging tasks (figure 3) show individuals solving both options at fairly regular intervals rather than in collective bursts, suggesting that solvers were not influenced by other solvers. Both estimates from the NBDA and support for each variable are shown in table 4.

**Table 4.** Two option NBDA results, showing support for factors affecting task solving by individuals that have discovered but not previously solved the task.

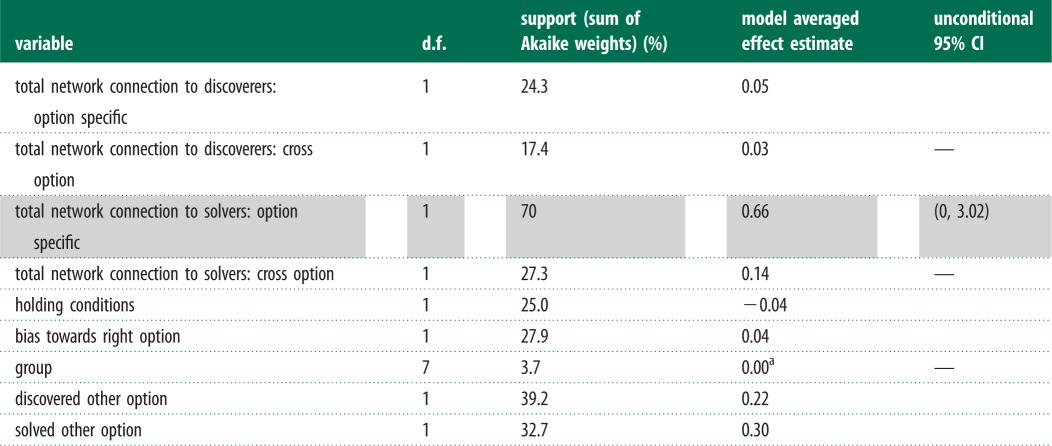

^a^Model averaged estimate of the difference between the fastest and slowest group.

There is some suggestive evidence of an option-specific effect of solvers (Akaike weight = 70%; *s* = 0.66; 95% CI (0, 3.02)), with individuals being more likely to solve a specific option if they were well connected to previous solvers of that option. However, the evidence is not strong, with the unconditional 95% CI including 0. Further, there was little evidence of an effect of familiar fish that had discovered either option, nor of familiar fish that had solved the other option (table 4).

## 4. Discussion

Our results provide evidence of an effect of familiarity upon group social organization, with fish being more likely to associate with familiar than with unfamiliar individuals. Furthermore, familiarity was seen to affect the likelihood of an individual discovering a foraging task, with strong evidence of a social effect on discovery of the foraging tasks, such that individuals tended to discover a task sooner if a familiar individual from their group had previously done so. Despite finding that familiarity affected both group social structure and the likelihood of an individual discovering the task, we found that the overall association network of a group had little effect upon the likelihood of an individual discovering a feeding task. In other words, a given fish was no more likely to discover the feeding task if it was strongly connected to fish that had previously discovered it than it was if it was poorly connected to previous discoverers. Finally, we found no evidence that reduced latency to discovery is a result of task location being socially transmitted between individuals, because the social effect on ‘discovery’ of an empty control patch was plausibly as strong as was discovery of a patch where foraging was possible. The marked similarity in social effects on foraging and control patches suggests that an untransmitted social effect might underlie the observed diffusions, almost certainly due to the influence of fish on one another's movements.

Such an interpretation is consistent with results of studies of foraging behaviour in stickleback shoals reported by Atton *et al*. [29] and for the ‘open environment’ condition by Webster *et al*. [30]. In both these studies conducted in open or relatively unstructured environments, similar to the one used in this study, network structure was a poor predictor of patch discovery [29,30]. By contrast, in structurally complex environments, order of patch discovery was linked to network structure [30]. This difference in outcome may be due to the effects of the environment upon network characteristics, as groups in structurally complex environments had lower network density overall, and more heterogeneous, ‘cliquey’ networks than groups in structurally simple environments. Difference in outcome may also be due to structured environments limiting the distance over which social cues indicative of patch location, such as feeding behaviours, can be detected, making it more likely that only closely associating individuals will detect cues containing foraging information from one another [29,30]. The size of the arena is likely to be important too, as this influences opportunities for dispersal and the frequency with which subgroups are likely to encounter one another.

In addition to influencing task discovery, familiarity was also seen to have an effect upon latency to solve the feeding tasks, with individuals being more likely to solve a task when familiar than when unfamiliar individuals had already done so. Evidence of a difference in the effects of familiar and unfamiliar individuals on task solution is not particularly strong. However, the familiarity network explains the data better than does a homogeneous network, suggesting a familiarity-based social effect is present. Such an interpretation is consistent with the social effects seen in the same model, where there was also some support for an effect on the rate of an individual solving a specific task when they were well connected to familiar previous solvers.

Finding an effect of familiarity on task solution is consistent with the results of previous studies, which have found that associations between familiar individuals can give rise to directed social transmission of information [20,23]. If so, shoaling with familiar fish may be adaptive, in that it may allow individuals to locate resources more rapidly or with greater efficiency when foraging with familiar than with unfamiliar group mates. Shoaling with familiar individuals has also been suggested to provide anti-predator benefits in some species through greater shoal cohesion [45].

The mechanism by which familiarity affects behaviour, and ultimately information acquisition, is not fully clear. Familiarity may reflect a perceptual or attentional bias for observing or more strongly responding to the behaviour of familiar individuals. Where familiarity is based upon diet- or habitat-derived cues, selection might favour behaviour whereby individuals follow or copy others that are exploiting the same range of resources [13,15]. A tendency to follow or copy the behaviour of individuals exploiting similar resources may also occur when familiarity is based solely upon learned recognition, because the development of learned recognition requires prolonged interaction that may also result in individuals being exposed to and exploiting similar resources. Our methods allowed both learned and resource-derived familiarity to develop within our treatment subgroups. Useful further work might seek to determine the relative contributions of these two forms of recognition to the effects observed in this study and to identify the way in which each increases the likelihood of patch discovery.

In summary, familiarity between shoal members had a clear effect on discovery of prey patches. Familiarity was also seen, to a lesser extent, to affect solving of a novel foraging task. These results demonstrate that factors that affect fine-scale social interactions can influence how individuals encounter and exploit resources, and suggest that researchers should take into account such social factors when investigating how information and behaviour diffuse through populations.

## Supplementary Material

NBDA further details

## Notes

This research was not judged to require a project licence from the UK Home Office. We adhered to all institutional regulations and national laws governing the use of animals in research. No fish died or showed any signs of injury or illness during or immediately after the study. Following the completion of this study, the fish were retained in the laboratory to be used in further research projects.

## Data accessibility

The dataset upon which this article is based can be accessed from Dryad (http://datadryad.org/, doi:10.5061/dryad.1mq62) [46].
