# Supplementary material for: Familiarity affects social network structure and discovery of prey patch locations in foraging stickleback shoals
Source: Proc Biol Sci. 2014 Aug 22;281(1789):20140579. doi: 10.1098/rspb.2014.0579 (PMC4100505; doi:10.1098/rspb.2014.0579)
Supplement: NBDA further details [file rspb20140579supp1.pdf]

Supplementary Material for

Familiarity affects social network structure and social transmission of prey patch locations in foraging stickleback shoals

Atton, N., Galef, B.J., Hoppitt, W., Webster, M.M. & Laland K.N.

1. Network based diffusion analysis (NBDA) model specification

The standard NBDA can be expressed, for multiple diffusions in different groups, as either:

$$\lambda_{ik}(t) = \lambda_{0k}(t)\big(1 - z_{ik}(t)\big)\left(s\sum_{j=1}^{N_k} a_{ijk} z_{jk}(t) + exp(\psi_{ik}) \right)$$

Eqn. S1a

In which the rate of social transmission and asocial learning combine additively, or a multiplicative model:

$$\lambda_{ik}(t) = \lambda_{0k}(t)\big(1 - z_{ik}(t)\big)\left(s\sum_{j=1}^{N_k} a_{ijk} z_{jk}(t) + 1 \right) exp(\psi_{ik})$$

Eqn. S1b

Where:
$\lambda_{ik}(t)$is the rate of acquisition (or "hazard rate" in survival analysis terminology) of the trait for individual *i* in group *k* at time *t;*
$\lambda_{0k}(t)$ is a baseline rate function for individuals in group *k*;
$z_{ik}(t)$ is an indicator variable giving the status of *i* in group *k* at time *t* (1= informed; 0=naïve);
a_{ijk} is the network connection from *j* to *i* in group *k*;
s is a parameter giving the strength of social learning (≥ 0; but see section 3, below); and
ψ_{ik} is a linear predictor allowing the modelling of the effect of individual level variables (e.g. size) in a manner analogous to a Cox survival model or generalised linear model (GLM); and
N_k is the number of individuals in group *k*.

OADA makes no assumptions about the shape of $\lambda_{0k}(t)$, and should therefore be used if a suitable model is not available for $\lambda_{0k}(t)$ (Hoppitt & Laland, 2011). We decided to use OADA since it makes fewer assumptions about the baseline acquisition function, though modified as described in section 2. The form of OADA for multiple diffusions given by Hoppitt et al. (2010) is only sensitive to the order of acquisition within groups, and so is not sensitive to between group patterns, i.e. it allows for a different $\lambda_{0k}(t)$ for each group. However, we felt it was reasonable to assume that there is a common baseline rate function for all groups $\lambda_{0k}(t) = \lambda_0(t)$, since groups were selected from the same population and diffusions run in identical and constant laboratory conditions. We accomplished this by treating the data the order across groups, effectively as a single diffusion, but with zero social network connections between individuals in different groups.

Atton et al (2012) expanded the simple NBDA model given above to a) allow for two options for solving the task, in this case solving either the left or right version of the task, and b) to separate effects on the rate at which individuals first discover each task and the rate at which they subsequently first solve each task. The standard NBDA is a two state model, with individuals moving from a naïve to an informed state (naïve->informed). In the expanded model used here, for each option fish are in one of three states, being naïve, having discovered that option but not solved it, or having solved that option. This allowed us to examine the social effects on each transition (naïve-> discovered and discovered non-solver -> solver). Analysing the rate of discovery (naïve->discovered) and solving (naïve -> solver) separately (e.g. Hoppitt et al 2010) fails to fully tease apart the two transitions. The additive two option model for discovery is expressed generally as follows:

The full additive models for the two-option extension are:

$$\lambda_{D,ik1}(t) = \lambda_{D,0k}(t)\big(1 - d_{ik1}(t)\big)\left(s_{DD,OS}\sum_{j=1}^{N_k} a_{ijk}d_{jk1}(t) + s_{DS,OS}\sum_{j=1}^{N_k} a_{ijk}z_{jk1}(t) + s_{DD,CO}\sum_{j=1}^{N_k} a_{ijk}d_{jk2}(t) + s_{DS,CO}\sum_{j=1}^{N_k} a_{ijk}z_{jk2}(t) + exp\left(\psi_{D,ik} + \alpha d_{jk2}(t) + \beta z_{jk2}(t)\right)\right)$$

Eqn. S2a

$$\lambda_{D,ik2}(t) = \lambda_{D,0k}(t)\big(1 - d_{ik2}(t)\big)\left(s_{DD,OS}\sum_{j=1}^{N_k} a_{ijk}d_{jk2}(t) + s_{DS,OS}\sum_{j=1}^{N_k} a_{ijk}z_{jk2}(t) + s_{DD,CO}\sum_{j=1}^{N_k} a_{ijk}d_{jk1}(t) + s_{DS,CO}\sum_{j=1}^{N_k} a_{ijk}z_{jk1}(t) + exp\left(\psi_{D,ik} + \alpha d_{jk1}(t) + \beta z_{jk1}(t)\right)\right)$$

Eqn. S2b

$\lambda_{D,ik1}(t)$ is the rate of discovery of option 1 and $\lambda_{D,ik2}(t)$ is the rate of discovery of option 2 and $\lambda_{D,0k}(t)$ the corresponding baseline function, taken here to be $\lambda_{D,0k}(t) = \lambda_{D,0}(t)$, see above.

s terms are parameters giving the various social effects tested for: subscript *DD* denotes on effect of discoverers on the rate of discovery, and *SD* the effect of solvers on discovery rate; the subscript *OS* indicates that a social effect is option specific, whereas *CO* indicates that it operates across options ("cross option"). e.g. $s_{DD,OS}$ is then a parameter giving the social effect of connected individuals who have discovered the same option on rate of discovery, whereas $s_{SD,CO}$ gives the social effect of connected individuals who have solved the different option on rate of discovery.

$\psi_{S,ik}$is a linear predictor as above;

$z_{jkm}(t)$ is an indicator for whether *i* in group *k* has solved the task using option *m* prior to time *t*;
$d_{ikm}(t)$ is an indicator for whether *i* in group *k* has discovered option *m* prior to time *t*, regardless of whether *i* has yet solved the task using option *m*;
α is the effect on discovery rate of option *m*, on the scale of the linear predictor, of having previously discovered the other option;
β is the effect on discovery rate of option *m*, on the scale of the linear predictor, of having previously solved the other option;

For all NBDA models, we suggest that individual-level variables included in the linear predictor, $\psi_{D,ik}$, are transformed by subtracting the mean across all individuals. This means that the social effects can be interpreted consistently as the increase in rate per unit of network connection, relative to the average asocial rate.

The rate of solving for each option is then given as follows:

$$\lambda_{S,ik1}(t) = \lambda_{S,0k}(t)\big(d_{ik1}(t) - z_{ik1}(t)\big)\left(s_{SD,OS}\sum_{j=1}^{N_k} a_{ijk}d_{jk1}(t) + s_{SS,OS}\sum_{j=1}^{N_k} a_{ijk}z_{jk1}(t) + s_{SD,CO}\sum_{j=1}^{N_k} a_{ijk}d_{jk2}(t) + s_{SS,CO}\sum_{j=1}^{N_k} a_{ijk}z_{jk2}(t) + exp\left(\psi_{S,ik} + \gamma d_{jk2}(t) + \delta z_{jk2}(t)\right)\right)$$

Eqn. S2c

$$\lambda_{S,ik2}(t) = \lambda_{S,0k}(t)\big(d_{ik2}(t) - z_{ik2}(t)\big)\left(s_{SD,OS}\sum_{j=1}^{N_k} a_{ijk}d_{jk2}(t) + s_{SS,OS}\sum_{j=1}^{N_k} a_{ijk}z_{jk2}(t) + s_{SD,CO}\sum_{j=1}^{N_k} a_{ijk}d_{jk1}(t) + s_{SS,CO}\sum_{j=1}^{N_k} a_{ijk}z_{jk1}(t) + exp\left(\psi_{S,ik} + \gamma d_{jk1}(t) + \delta z_{jk1}(t)\right)\right)$$

Eqn. S2d

where:
$\lambda_{S,ikm}(t)$ is the rate of solving of option *m* by *i* in group *k* at time *t* and $\lambda_{S,0k}(t)$ the corresponding baseline function, taken here to be $\lambda_{S,0k}(t) = \lambda_{S,0}(t)$, see above.
On s parameters, *DS* denotes the effect of discoverers on solving rate; the subscript *SS* denotes the effect of solvers on solvers, on *OS*/*CO* denote option-specific and cross-option effects as above;
γ is the effect on discovery rate of option *m*, on the scale of the linear predictor, of having previously discovered the other option;

δ is the effect on discovery rate of option m, on the scale of the linear predictor, of having previously solved the other option.

The common baseline rate functions across options, $\lambda_{D,0k}(t)$ and $\lambda_{S,0k}(t)$ are taken to be $\lambda_{D,0}(t)$ and $\lambda_{S,0}(t)$ respectively (the same shape across groups). This means that the model for discovery takes as data the order of discovery across groups and options, and the model for solving takes as data the order of solving across groups and options. The $\left(d_{ikm}(t) - z_{ikm}(t)\right)$ term ensures individuals are only included in the likelihood function for a solving event at time *t* for options they have discovered, $(d_{ikm}(t) = 1)$ but not solved $(z_{ikm}(t) = 0)$.

We calculated the likelihood function in an analogous manner to the standard OADA (see Hoppitt et al 2010a) or Cox model (Therneau & Grambsch 2000). Models were fitted using maximum likelihood using the nlminb or optim optimisation functions in the R 2.15.3 statistical environment (R Core Team 2013).

The full multiplicative models are given by:

$$\lambda_{D,ik1}(t) = \lambda_{D,0k}(t)\big(1 - d_{ik1}(t)\big)\left(s_{DD,OS} \sum_{j=1}^{N_k} a_{ijk} d_{jk1}(t) + s_{DS,OS} \sum_{j=1}^{N_k} a_{ijk} z_{jk1}(t) + s_{DD,CO} \sum_{j=1}^{N_k} a_{ijk} d_{jk2}(t) + s_{DS,CO} \sum_{j=1}^{N_k} a_{ijk} z_{jk2}(t) + 1 \right) exp\left(\psi_{D,ik} + \alpha d_{jk2}(t) + \beta z_{jk2}(t)\right)$$

Eqn. S3a

$$\lambda_{D,ik2}(t) = \lambda_{D,0k}(t)\big(1 - d_{ik2}(t)\big)\left(s_{DD,OS} \sum_{j=1}^{N_k} a_{ijk} d_{jk2}(t) + s_{DS,OS} \sum_{j=1}^{N_k} a_{ijk} z_{jk2}(t) + s_{DD,CO} \sum_{j=1}^{N_k} a_{ijk} d_{jk1}(t) + s_{DS,CO} \sum_{j=1}^{N_k} a_{ijk} z_{jk1}(t) + 1 \right) exp\left(\psi_{D,ik} + \alpha d_{jk1}(t) + \beta z_{jk1}(t)\right)$$

Eqn. S3b

$$\lambda_{S,ik1}(t) = \lambda_{S,0k}(t)\big(d_{ik1}(t) - z_{ik1}(t)\big)\left(s_{SD,OS} \sum_{j=1}^{N_k} a_{ijk} d_{jk1}(t) + s_{SS,OS} \sum_{j=1}^{N_k} a_{ijk} z_{jk1}(t) + s_{SD,CO} \sum_{j=1}^{N_k} a_{ijk} d_{jk2}(t) + s_{SS,CO} \sum_{j=1}^{N_k} a_{ijk} z_{jk2}(t) + 1 \right) exp\left(\psi_{S,ik} + \gamma d_{jk2}(t) + \delta z_{jk2}(t)\right)$$

Eqn. S3c

$$\lambda_{S,ik2}(t) = \lambda_{S,0k}(t)\big(d_{ik2}(t) - z_{ik2}(t)\big)\left(s_{SD,OS} \sum_{j=1}^{N_k} a_{ijk} d_{jk2}(t) + s_{SS,OS} \sum_{j=1}^{N_k} a_{ijk} z_{jk2}(t) + s_{SD,CO} \sum_{j=1}^{N_k} a_{ijk} d_{jk1}(t) + s_{SS,CO} \sum_{j=1}^{N_k} a_{ijk} z_{jk1}(t) + 1 \right) exp\left(\psi_{S,ik} + \gamma d_{jk1}(t) + \delta z_{jk1}(t)\right)$$

Eqn. S3d

2. Model averaging and unconditional confidence intervals

Instead of using a model-selection procedure to choose a best model, we used a model averaging approach, using Akaike's Information Criterion, corrected for sample size (AICc) (Burnham and Anderson, 2002). Inferences based on model averaging take into account uncertainty as to which model is best. AICc estimates the Kullback-Leibler (K-L) information for a model: the extent to which the predicted distribution for the dependent variable approximates its true distribution). The AICc allows us to calculate an Akaike weight for each model that gives the probability that the model is the actual best K-L model (that with the lowest K-L information) out of those considered, allowing for sampling variation. By summing Akaike weights for all models that include a specific variable, we obtain the probability that a variable is in the best K-L model, thus quantifying support the data give for an effect of a variable.

This approach is preferable to calculating a p value to quantify the strength of evidence for each effect, because: (i) the p value depends on which model is chosen and consequently does not account for model uncertainty, and (ii) a large p value tells us little about the strength of evidence against an effect (i.e. whilst statistical power can be calculated, the power has to be for a specified, usually arbitrary, effect sizes). Therefore, for each variable considered, we give its total Akaike weight (as a %) and model averaged estimate. We also provide unconditional 95% confidence intervals using Burnham and Anderson's method for adjusting profile likelihood confidence intervals for model selection uncertainty. The details of the procedure we used are as follows:

We calculated model-averaged estimates for each parameter using the procedure given in Burnham and Anderson (2002). We performed model averaging across additive and multiplicative social transmission models separately, since the s parameters operate on different functions in each case. In every case we calculated across the entire set of such models, since, where are parameter was not present it could be interpreted as having a value of zero (Burnham & Anderson 2002).

Burnham and Anderson (2002) present a method for calculating unconditional standard errors for parameters that allows for model uncertainty, which can be used to calculate unconditional Wald confidence intervals. However, we found this method to be misleading since standard errors only reflect the local shape of the likelihood function at the maximum likelihood estimator (MLE), and so Wald confidence intervals can be misleading where the likelihood surface if asymmetrical about the MLE. This is the case for many of our parameters.

95% confidence intervals can also be constructed using profile likelihood techniques (Morgan 2009), which involves determining the set of values for a parameter, θ, that could not be rejected at the 5% level in a likelihood ratio test (LRT) against a model in which θ is unconstrained. This gives a more accurate picture when the likelihood surface is asymmetrical. Burnham and Anderson (2002) propose a method for adjusting profile likelihood confidence intervals by increasing the critical value for rejection in the LRT, based on the unconditional standard error. We calculated profile likelihoods from the best K-L model and used obtained conditional standard errors for each model from the numerical estimate of the Hessian matrix, returned by the optim function.

Additional references not cited in main text

Burnham, K.P. and Anderson, D.R. 2002. *Model selection and Multimodel Inference*. New York: Springer-Verlag.

Hoppitt, W. and Laland, K.N. 2011. Detecting social learning in networks: A user's guide. *Am. J. Primatol*. 73: 834-844.

Morgan, B.J.T. (2009) Applied Stochastic Modelling. 2nd edition. Boca Raton: Chapman & Hall/ CRC Press.

Therneau, T.M. & Grambsch, P.M. (2000) Modeling survival data: extending the Cox model. New York: Springer.
